# A Novel Computer-Aided Diagnosis Scheme on Small Annotated Set: G2C-CAD

**DOI:** 10.1155/2019/6425963

**Published:** 2019-04-15

**Authors:** Guangyuan Zheng, Guanghui Han, Nouman Q. Soomro, Linjuan Ma, Fuquan Zhang, Yanfeng Zhao, Xinming Zhao, Chunwu Zhou

**Affiliations:** ^1^Beijing Key Laboratory of Intelligent Information Technology, School of Computer Science and Technology, Beijing Institute of Technology, Beijing 100081, China; ^2^School of Information and Technology, Shangqiu Normal University, No. 55 Pingyuan Road, Shangqiu, Henan Province, China; ^3^School of Biomedical Engineering, Sun Yat-sen University, Guangzhou 510006, China; ^4^Department of Software Engineering, Mehran University of Engineering and Technology, SZAB Campus, Khairpur Mirs 76062, Pakistan; ^5^Fujian Provincial Key Laboratory of Information Processing and Intelligent Control, Minjiang University, Fuzhou 350121, China; ^6^Digital Performance and Simulation Technology Lab., School of Computer Science and Technology, Beijing Institute of Technology, 100081 Beijing, China; ^7^Department of Imaging Diagnosis, Cancer Institute and Hospital, Chinese Academy of Medical Sciences, Beijing 100021, China

## Abstract

**Purpose:**

Computer-aided diagnosis (CAD) can aid in improving diagnostic level; however, the main problem currently faced by CAD is that it cannot obtain sufficient labeled samples. To solve this problem, in this study, we adopt a generative adversarial network (GAN) approach and design a semisupervised learning algorithm, named G2C-CAD.

**Methods:**

From the National Cancer Institute (NCI) Lung Image Database Consortium (LIDC) dataset, we extracted four types of pulmonary nodule sign images closely related to lung cancer: noncentral calcification, lobulation, spiculation, and nonsolid/ground-glass opacity (GGO) texture, obtaining a total of 3,196 samples. In addition, we randomly selected 2,000 non-lesion image blocks as negative samples. We split the data 90% for training and 10% for testing. We designed a DCGAN generative adversarial framework and trained it on the small sample set. We also trained our designed CNN-based fuzzy Co-forest on the labeled small sample set and obtained a preliminary classifier. Then, coupled with the simulated unlabeled samples generated by the trained DCGAN, we conducted iterative semisupervised learning, which continually improved the classification performance of the fuzzy Co-forest until the termination condition was reached. Finally, we tested the fuzzy Co-forest and compared its performance with that of a C4.5 random decision forest and the G2C-CAD system without the fuzzy scheme, using ROC and confusion matrix for evaluation.

**Results:**

Four different types of lung cancer-related signs were used in the classification experiment: noncentral calcification, lobulation, spiculation, and nonsolid/ground-glass opacity (GGO) texture, along with negative image samples. For these five classes, the G2C-CAD system obtained AUCs of 0.946, 0.912, 0.908, 0.887, and 0.939, respectively. The average accuracy of G2C-CAD exceeded that of the C4.5 random decision tree by 14%. G2C-CAD also obtained promising test results on the LISS signs dataset; its AUCs for GGO, lobulation, spiculation, pleural indentation, and negative image samples were 0.972, 0.964, 0.941, 0.967, and 0.953, respectively.

**Conclusion:**

The experimental results show that G2C-CAD is an appropriate method for addressing the problem of insufficient labeled samples in the medical image analysis field. Moreover, our system can be used to establish a training sample library for CAD classification diagnosis, which is important for future medical image analysis.

## 1. Introduction

Pulmonary carcinomas are the most lethal disease in the world. Approximately 1.5 million people die due to pulmonary cancer every year—far higher than the mortality rate of other diseases [1]. In the United States alone, the lung cancer deaths in 2018 will reach 154,050 [2]. However, most pulmonary tumors cause no symptoms. Therefore, the disease is usually diagnosed at advanced stages, resulting in a low overall 5-year survival rate of approximately 14% [3]. In contrast, the 5-year survival rate of patients with stage IA non-small cell lung cancer that has been pathologically confirmed and resected or precision treated [4–6] can reach 83% [7–9]. Thus, early lung cancer detection can sharply decrease the lung cancer mortality rate [10, 11]. Pulmonary primary cancers manifest as nodules in the early stage. Compared to chest X rays, computed tomography (CT) has shown higher sensitivity in detecting small lung nodules [3, 12]. Currently, CT screening is the most recommended method for finding nodules [13, 14]. The associated increase in spiral CT screening has led to a growing burden on radiologists [15]. Despite the higher resolution available today, it is still difficult for radiologists to distinguish malignant nodules from benign ones in low-dose CT (LDCT) images. The rates of resected benign pulmonary nodules can reach 50% during surgery [16–19]. These unnecessary surgeries cause physical and mental pain and impose additional financial burdens on patients.

A “sign” in a CT lung scans refers to a radiologic finding that suggests a specific disease process. Understanding the meaning of a sign implies an understanding of the findings on the CT scan [20]. Signs are also called “CT features,” “CT manifestation,” “CT patterns,” or sometimes “CT findings” [21]. Lobulation signs [22, 23], spiculation signs [15, 24–28], and some texture signs [29, 30] play crucial roles in radiologists' ability to differentiate benign from malignant nodules [31, 32]. Noncentral calcification, such as punctate sign or eccentric sign calcification, usually indicates that a nodule is malignant [33–35]. Therefore, it is highly important to study methods for identifying the signs of pulmonary nodules automatically to assist radiologists in diagnosing pulmonary malignant nodules.

One of the main methods is using Computer-Aided Detection/Diagnosis (CAD). The earliest conception of CAD appeared in the 1960s [36, 37]. Its early idea was attempting to “fully automate the chest exam.” Over the decades, this expectation has subsided (which seems to have happened to the early enthusiasm regarding the capabilities of artificial intelligence systems in general). Currently, the general agreement is that the focus should be on making useful computer-generated information available to physicians for decision support rather than trying to make a computer act like a diagnostician [38]. Various works on the CAD based on CT signs have been published. Han G et al. [22] designed a sliding-window-based framework to detect lobulation sign. Suzuki K et al. [39] developed a computer-aided diagnostic (CAD) scheme to distinguish benign from malignant nodules in LDCT scans using a massive training artificial neural network (MTANN) and concluded that spiculation sign is a highly differentiated feature for distinguishing benign from malignant nodules. CAD systems based on texture sign features have been investigated in several studies [40–42]. Since AlexNet won the ImageNet challenge by a large margin in 2012, deep learning techniques have flourished rapidly in the image detection field. Compared to traditional classification algorithms, deep learning can extract most distinctive features automatically and can implement end-to-end operations. However, the size of the dataset required to train a high capacity deep learning framework is quite large, while generating labeled training data in the medical image analysis field is very expensive [43].

To address the dilemma of having only a small annotation set available for lung nodule sign recognition, in this paper, we propose a semisupervised generative adversarial network (GAN) and a convolutional neural network (CNN)-based Co-forest CAD scheme. We apply the designed scheme to classify four types of nodule signs that are highly related to lung cancer. We call this proposed scheme G2C-CAD in abbreviation.

**Figure 1:**
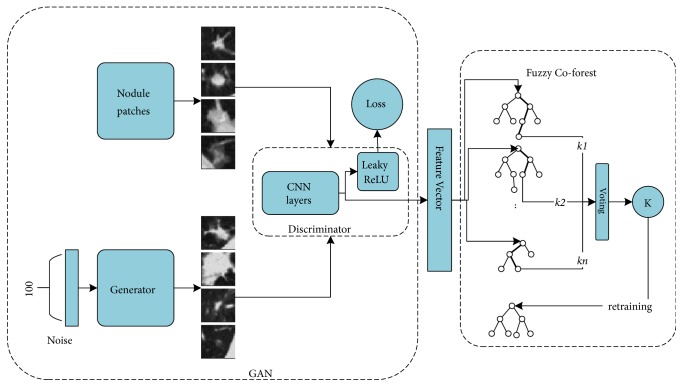
GAN- and CNN-based Co-forest computer aided diagnosis (G2C-CAD) system workflow.

Overall workflow of G2C-CAD is illustrated in Figure 1. In the stage A, we train a GAN and a Co-forest on the available small sample set. Then, in stage B, we use the trained GAN to generate a synthetic nodule patch and transfer it to the trained CNN discriminator. From the discriminator, we gain the CNN-extracted features of the synthesized nodule patch. In stage C, the CNN features are provided to the fuzzy Co-forest pretrained on the original sample set to conduct semisupervised learning for the five types of ROI patches. Finally, the process iterates between stages B and C until a termination condition is met.

The rest of this paper is organized as follows. In Section 2, we review the existing lung nodule classification algorithms.  Section 3 presents our G2C-CAD algorithm. We introduce the experimental method in Section 4 and present an analysis in Section 5.  Section 6 concludes this paper.

## 2. Related Work

Discriminating between benign or malignant nodules has attracted the interest of a large number of researchers. The early discrimination methods were based primarily on traditional machine learning algorithms such as k-nearest neighbors (k-NN), linear discriminant analysis (LDA), Bayes [44], rule based schemes, decision trees (DT), and the support vector machine (SVM). Krewer et al. [45] tested several classifiers, including DT, k-NN, and SVM, on extracted texture and shape features to discriminate malignant from benign nodules. By analyzing the experimental results, they found that partly solid and nonsolid nodules have a higher malignancy rate than do solid nodules. Colin Jacobs et al. [46] developed and evaluated a computer aided diagnostic system for classifying lung nodules into solid, partly solid, and nonsolid nodules. This CADx system performs statistical classification on the nodules' intensity-, texture-, and segmentation-based features using the k-NN algorithm. Xiabi Liu et al. [47] proposed a feature selection method based on the FIsher criterion and genetic optimization (FIG) to address Common CT Imaging Signs of Lung (CISL) disease recognition problems. They applied the FIG feature selection algorithm to bag-of-visual-words features, wavelet transform-based features, the Local Binary Pattern, CT Value Histogram features, and others. Their results showed that FIG achieved high computational efficiency and was highly effective. Tao Sun et al. [48] investigated an SVM-based CADx system for lung cancer classification using a total of 488 input features that included textural features, patient characteristics, and morphological features to train the classifier. Hidetaka Arimura et al. [49] developed a computerized scheme to automatically detect lung nodules in LDCT images for lung cancer screening. They extracted possible nodule images using a ring average filter, identified a set of nodule candidates by applying a multiple-gray-level thresholding technique, and removed false positives by using two rule-based schemes on the localized image features related to morphology and gray levels. Tao Sun et al. [50] proposed a CADx system to predict the characteristics of solitary pulmonary nodules in lung CT to diagnose early stage lung cancer. In their CADx system an SVM model was constructed that exploited curvelet transform texture features, 3 patient demographic features, and 9 morphological features. Fangfang Han et al. [51] constructed a CADx system based on 50 categories of 3D textural features extracted from gray levels, a curvature cooccurrence matrix, and gradients as well as other nodule volume data derivatives.

The above CAD systems mainly utilized features obtained by traditional feature extraction algorithms. Traditional feature acquisition is based on manual design and selection, which requires experts with specialized heuristic knowledge. The features obtained in this way are low-level features near the pixel level, and the work scope is relatively small. In the image analysis workflow, the final performance of the system also depends on the quality of prior preprocessing or segmentation stages. Therefore, in the traditional CAD solutions, tuning the classification performance is both complicated and arduous [52].

The emergence of the convolutional neural network (CNN) [53] solved this dilemma. In 2012, the emergence of AlexNet sparked a revival in the image detection field through deep learning techniques based on CNN features, and CNNs have subsequently been used extensively in pulmonary imaging analysis. Compared to traditional algorithms, CNNs can extract more distinctive features automatically. Two studies [52, 54] demonstrated that CNNs are a promising technique in lung nodule identification. Deep learning techniques have the inherent superiority of being able to automatically extract features and adjust the performance seamlessly.

In traditional CAD algorithms, training only needs to seek an optimal discriminant surface from the manually designed feature space. In contrast, deep learning networks simultaneously attempt to find both the most significant discriminate features among large numbers of high-level features and an optimal classification surface. As a result, training a deep learning network requires a massive amount of labeled samples [55]—a requirement that cannot be met in the medical image analysis field because professional annotation is too expensive [56]. Data augmentation techniques produce only limited effects. Although transfer learning can alleviate the problem of the lack of training examples to a certain extent, the significant feature sequences vary between different classification tasks. Thus, transfer learning is not the most suitable method to cope with medical image analysis tasks.

Generative adversarial networks (GANs) [57] have demonstrated the promising ability to generate visually realistic images. A GAN trained with limited annotated samples can generate large numbers of realistic images. Chuquicusma MJ et al. [58] conducted visual Turing tests to evaluate the degree of realism in nodule images generated by a DCGAN and showed that the generated samples can be used to boost the diagnostic power by mining high-level discriminative image features and that the resulting features can be used to train both radiologists and deep networks.

Semisupervised learning is a type of machine learning method that combines supervised and unsupervised learning. It can be applied when only a small number of labeled data exist, but a large number of unlabeled data are available. Semisupervised learning is important for reducing the cost of acquiring labeled data and improving classifier performance. Commonly used methods include EM with generative mixture models, self-training, cotraining, transductive support vector machines, and graph-based methods [59]. Co-forest is a cotraining based semisupervised learning algorithm that first learns an initial classifier from a small amount of labeled data and then refines the classifier by further exploiting a larger number of unlabeled data to boost the classifier's performance. When applying micro calcification detection for breast cancer diagnosis, Ming Li et al. [60] showed that Co-forest can successfully enhance the performance of a model trained on only a small amount of diagnosed samples by utilizing the available undiagnosed samples.

The above works inspired us to exploit a GAN to enlarge and enrich a training set of pulmonary nodules. It also motivated us to implement the designed G2C-CAD.

## 3. Materials and Methods

### 3.1. Experimentation Materials

**Figure 2:**
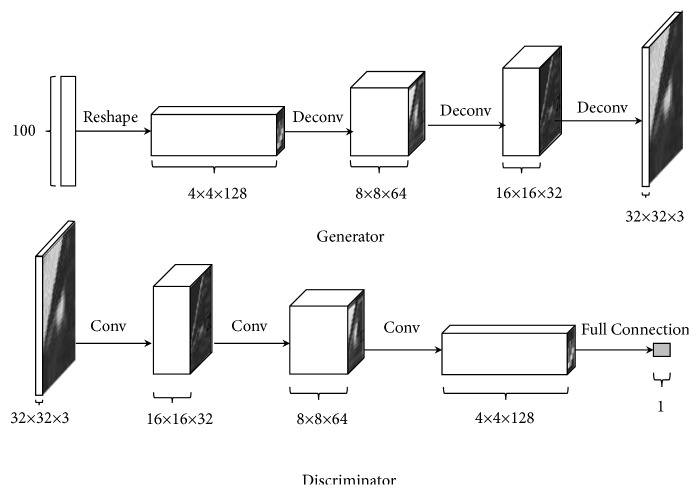
The architecture of the generator and the discriminator in the DCGAN model.

Insufficient labeled samples represents a barrier to CAD progress. The emergence of GAN [57] begun to change this situation. A GAN consists of two main parts: a generator G and a discriminator D. The G is used to learn the distribution of real images. Then it generates realistic images to attempt to fool the D. The D attempts to perform true and false discriminations concerning received images. Throughout the process, the G strives to make the generated image more realistic, while the D tries to identify the true and false images. This process is equivalent to a game with two opponents. Over time, the G and D eventually achieve a dynamic equilibrium in which images generated by the generator are highly similar to the real image distribution, and the discriminator cannot determine whether a sample is drawn from the true data or generated by the generator. DCGAN [61] is a GAN extension in which a CNN is introduced to conduct unsupervised training. The ability of the CNN to extract features is used to enhance the training of the generation network. Building on this idea, we constructed a 32 × 32 input scale DCGAN. The discriminator architecture in DCGAN has 4 layers, as shown in Figure 2.

**Figure 3:**
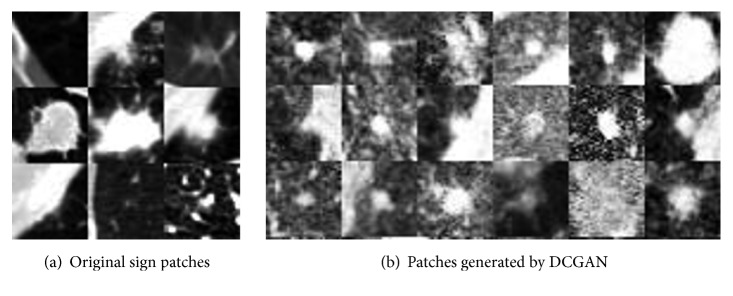
Samples of DCGAN-generated sign patches and real samples. The image on the left shows examples of the seed samples used for training. Most of the generated samples in (b) are realistic; although some of the generated patches can be recognized as being fake (the 5th one in row 3), they do help to boost the CAD system's performance.

We tested a GAN trained from 9 samples to generate unlabeled-nodule sign patches, and the result is gratifying, as shown in Figure 3.

### 3.2. CNN Feature-Based Fuzzy Co-Forest Method

**Figure 4:**
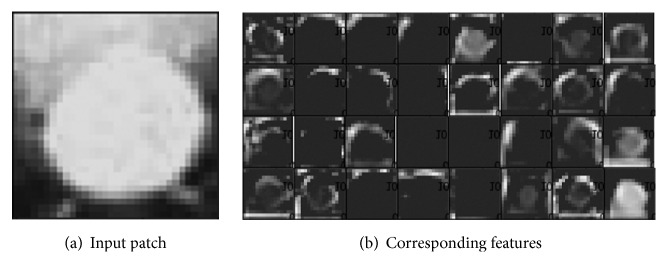
(a) An original input sign patch; (b) the corresponding 32 CNN features abstracted from the layer 1 of the discriminator D.

As discussed in Section 3.1, when we gain a trained GAN, we also get a trained CNN discriminator simultaneously. For each image patch passed though the discriminator, we obtain a 128-dimensional CNN feature vector from the last convolutional layer. Features of 4 × 4 is hard for eyes to discern; in Figure 4 we show an example of 32-dimensional 16 × 16 CNN features of a sign patch extracted from layer 1 of D.

Cotraining random forest (Co-forest) is an upgraded algorithm for the cotraining paradigm. The standard cotraining algorithm has two strong assumptions: (1) the samples distribution is consistent with that of the target functions and (2) the different features extracted from the same data should be conditionally independent. In most cases, however, these two strong assumptions are difficult to satisfy. Co-forest uses an ensemble consisting of multiple classifiers to avoid the constraints of standard cotraining. The specific structure of random forests enables the Co-forest to take advantage of semisupervised learning and ensemble learning to better learn the distribution of the training data. Existing Co-forest works are based on traditional manually designed features [60, 62]. Here, we try to extend the Co-forest approach to deep neural networks by utilizing the CNN features obtained from the GAN's discriminator.

For each generated realistic sign from DCGAN, we can extract a 128-element 4 × 4 CNN feature vector. Each 4 × 4 CNN feature can be transformed to a 1 × 16 vector. If we assume that DCGAN runs N times, then we will obtain N CNN feature vectors from the discriminator. These CNN feature vectors for the N image patches can be expressed by a matrix:

(1) Original_F=f1,0…f1,127f2,0…f2,127⋮fN,0…fN,127

(2) f=e0…e15,

where in (1)* f* is a 16-element vector transformed from a 4 × 4 feature patch. Every row in Original_F represents the 128-dimensional CNN features from one input image patch. The features in each column are produced from the same filter. The* n* in *f*_*n*,*m*_ represents the *nth* sign in N, and* m* represents the* mth* feature of a sign. We build a complete reference vector *r* = [1,1,…, 1], |*r*| = 16. The cosine similarity of any feature* f *to* r *can be calculated as* a*:

(3) $$\begin{array}{c} a=\frac{e_{0}*1+e_{1}*1+\cdots +e_{15}*1}{\sqrt{{e_{0}}^{2}+{e_{1}}^{2}+\cdots +{e_{15}}^{2}}\times \sqrt{1^{2}+1^{2}+\cdots +1^{2}}}, \end{array}$$

where *e*_*i*_ is an element of* f*. We use a distance matrix A of the relative cosine distances to* r* to replace the feature matrix Original_F:

(4) $$\begin{array}{c} A=\left[\begin{array}{ccc} a_{1,0}\ldots & & a_{1,127}\\ a_{2,0}\ldots & & a_{2,127}\\ & \vdots & \\ a_{N,0}\ldots & & a_{N,127} \end{array}\right]. \end{array}$$

From matrices (1) and (4), we can see that for any two elements *a*_i_ and *a*_j_, a smaller difference between the values of *a*_i_ and *a*_j_ indicates that their corresponding features *f*_i_ and *f*_j_ are more similar. The elements in each column of A are a series of continuous-valued data. To build a Co-forest, the first step is to construct decision trees by utilizing the labeled samples [63]. Assume that the total number of samples is N_L_ and that the classes are* c*_1_,* c*_2_,…, *c*_k_,* k*. To build a decision tree, we randomly select S samples. These S samples are sorted on A[*x*] by the values of *a*_i_, where x represents the* xth* column of A. We calculate the middle value between *a*_i,x_ and *a*_i+1,x_ as a split point *t*_ix_. The class information entropy of S is

(5) $$\begin{array}{c} Info\left(\;S\;\right)=-\overset{k}{\underset{j=1}{\sum }}p_{j}\;\log_{2}\left(\;p_{j}\;\right), \end{array}$$

where P_j_ represents the proportion of the category* j* samples relative to all samples, and S_j_ is a subset of S constructed by all the elements of S belonging to class c_j_, *p*_j_ = |S_j_|/|S|.

When selecting feature *t*_ix_ as the splitting node of the decision tree, the information entropy of S is

(6) InfoAx,tix:S=Sj<tixS×InfoSj<tix+Sj>tixS×InfoSj>tix.

Then, we compute the information gain:

(7) $$\begin{array}{c} Gain\left(\;A\left[\;x\;\right],t_{ix}:S\;\right)=Info\text{(S)-}Info\left(\;A\left[\;x\;\right],t_{ix}:S\;\right). \end{array}$$

The split information entropy is

(8) SplitAx,tix:S=−Sa<tixS×log2⁡Sa<tixS+Sa>tiS×log2Sa>tixS,

and the information gain ratio is

(9) $$\begin{array}{c} IGR=\frac{Gain\left(A\left[x\right],t_{ix}:S\right)}{Split\left(A\left[x\right],t_{ix}:S\right)}. \end{array}$$

In a column of attributes A[x], a suitable threshold *t*_max_ to divide A[x] into two intervals is A[x]_1_(*a*_i_ < *t*_max_, *a*_i_ ∈ A[x]_1_), A[x]_2_(*a*_i_ > *t*_max_, *a*_i_ ∈ A[x]_2_). This split produces the maximal information gain ratio. This *t*_max_ is then selected as the parent node to generate two children. This process is conducted recursively to build a decision tree until a termination criterion is matched.

**Figure 5:**
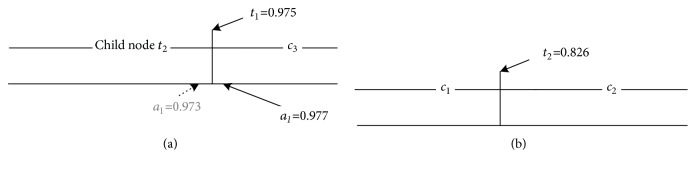
Traditional classification process.

In the reasoning process, if a sample's attribute value falls into a small region around *t*_ix_, after it is disturbed by noise, it can easily be misclassified. As shown in Figure 5, suppose we have a sample whose real attribute values are a1=0.977 and a2=0.827; however, due to noise during collection, a1 is changed to 0.973. In a traditional decision tree, this sample would be misclassified as *c*_2_.

**Figure 6:**
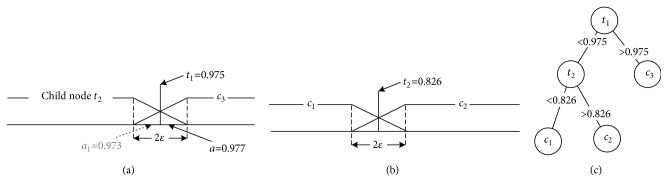
Here, (a) and (b) are fuzzy classification processes, and (c) is the decision tree.

To avoid this fragility, when constructing a decision tree, we utilize a fuzzy scheme [63]. In a traditional decision tree, when a sample's attribute* a* is greater than *t*_ix_, the sample belongs to either *c*_m_ or *c*_n_. We modify this crisp classifying method by selecting a neighborhood threshold* ε* around the split point *t*_ix_, as shown in Figures 6(a) and 6(b), so that the classification function becomes

(10) $$\begin{array}{c} C\left(\;s\;\right)=\left\{\;\frac{t_{i}+\varepsilon -a}{2\varepsilon }\times c_{m},\frac{{a-t}_{i}+\varepsilon }{2\varepsilon }\times c_{n}\;\right\}, \end{array}$$

where C(s) is a classification function that maps sample* s *to one of two weighted classes.

As shown in Figure 6(c), along one branch, the final classification *c*_i_ is calculated as follows:

(11) $$\begin{array}{c} c_{i}=\overset{m}{\underset{j=1}{\prod }}\left\{\begin{array}{cc} \frac{t_{j}+\varepsilon -a_{j}}{2\varepsilon }; & \text{left branch of }t_{j.}\\ \frac{a_{j}-t_{j}+\varepsilon }{2\varepsilon }; & \text{right branch of }t_{j.} \end{array}\right., \end{array}$$

where *c*_i_ is one class from {*c*_1_, *c*_2_,…, *c*_k_}.

When *ε* = 0.1, under the fuzzy classifying scheme, the classification result of disturbed example* s *is C(*s*) = {0.253*∗c*_1_, 0.258*∗c*_2_, 0.49*∗c*_3_}. It is obvious that the maximal probability of the final decision result is max⁡(C(s)) = *c*_3_. Based on this classification example, we can see that the fuzzy decision scheme is more robust to noise.

The classification result of the fuzzy Co-forest is a multiple-label probability distribution of the union of the decision trees' output; we consider the class with the highest probability as the final output.

Let LB denote the labeled set. G(*z*) denotes the process of generating a new fake sign-image patch utilizing the trained DCGAN. There are N classifiers in the Co-forest ensemble *E*^*∗*^. *e*_i_  (i = 1,…, N). We denote one of the classifiers in ensemble, *E*_i_, as the concomitant ensemble of *e*_i_ and create a subensemble that includes all the classifiers except *e*_i_. For a newly generated sample from G, if the max fuzzy vote sum of the classifiers in concomitant ensemble *E*_i_ exceeds a preset threshold *θ*, the sample will be copied to a newly labeled set *LB*_*i*_′ with the new assigned label. Based on [64], the process iterates until ${\hat{e}}_{i,t-1}W_{i,t-1}/{\hat{e}}_{i,t}$ is larger than *W*_*i*,*t*_.Then, the set *LB* ∪ *LB*_*i*,*t*_′ is used to refine *e*_i_. $\hat{e}$ denotes the classification error rate, and *W*_*i*,*t*_ = ∑_*j*=0_^*m*_*i*,*t*_^*w*_*i*,*t*,*j*_, where *w*_*i*,*t*,*j*_ is the predicted confidence of *E*_*i*_ on *LB*_*i*,*t*_′, and *m*_*i*,*t*_ is the size of set *LB*_*i*,*t*_′.

Based on the fuzzy decision tree scheme, we construct the fuzzy Co-forest as shown in Algorithm 1.

## 4. Experiments

### 4.1. Datasets

We collected sample instances from both the LIDC-IDRI and LISS datasets. LIDC-IDRI [65] consists of pulmonary medical image files (such as CT scans and X-rays) with corresponding pathological annotations. The data were collected by the National Cancer Institute to study early cancer detection in high-risk populations. LISS [21] consists a set of CISLs collected by the Cancer Institute and Hospital at the Chinese Academy of Medical Sciences and the Beijing Institute of Technology intended for computer-aided detection and diagnosis research and medical education. LISS contains 271 CT scans and 677 abnormal regions, including nine categories of CISLs.

### 4.2. LIDC-IDRI Instances

**Figure 7:**
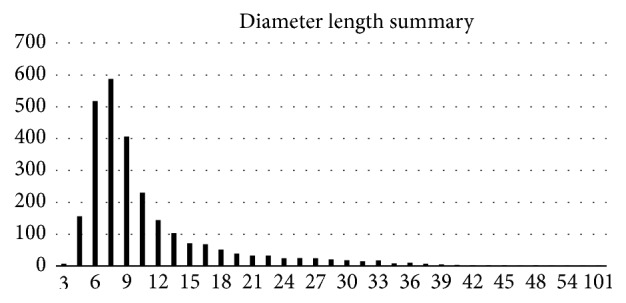
The diameter-size distribution of the nodules in the LIDC-IDRI dataset.

In the LIDC-IDRI CT imaging slices, most of the annotated nodules have diameters less than 32 pixels, as shown in Figure 7. Therefore, we choose 32 × 32 as the input ROI size.

A higher degree of lobulation, speculation, and nonsolid texture signs indicates a greater probability of malignant nodules [66]. A calcification sign usually indicates a benign nodule [66–68] except when it has a noncentral appearance. Signs of subtlety [69], internal structure [27], sphericity, and margin [33] have not been clearly proven to have a strong relationship with malignancy. To simplify the comparisons in this experiment, we selected nodules from LIDC with calcification =4 (noncentral calcification), lobulation >=4, spiculation >=4, texture <=2, and malignancy >=3 as experimental instances based on the selection rules shown in Table 1.

LIDC-IDRI contains 21,057 annotated nodules. We selected the 4 category nodules with signs such as noncentral calcification, lobulation, speculation, and nonsolid/GGO texture that have a high prevalence of malignancy as the experimental objects. For the probability of malignancy in these nodules, we adopted the average value of 4 radiologists' scores. The noncentral calcification, lobulation, spiculation, and nonsolid/GGO texture signs are illustrated in Table 2. In addition, we randomly extracted 32 × 32-pixel image patches from slices not annotated by any radiologist as negative samples. In total, we used five types of image blocks in our experiment.

Based on the center point of the merged regions annotated by 4 experts, we extracted 32 × 32-pixel image blocks as the experimental input by following the selection criteria shown in Table 1. Among these samples, we ensured that a given category of ROI patches for a single patient does not appear in any two subsets simultaneously, which helped ensure that the specificity of the trained individual networks is as high as possible.

### 4.3. LISS Instances

From LISS, we selected signs of lobulation, spiculation, pleural indentation, and GGO associated with malignant lung cancer [70–74] as the experimental objects. The number of samples in each category is shown in Table 3.

### 4.4. Evaluation Criteria

To evaluate the performance of the algorithm presented in this paper, we considered the following criteria.

1. ROC: The Receiver Operator Characteristic curve (ROC) is a method that comprehensively and simultaneously reflects the sensitivity and specificity of the classification result. By comparing the classification results of different samples with the annotation labels, a series of sensitivity and specificity scores is calculated. Then, a curve is drawn using sensitivity as the ordinate and 1 − specificity as the abscissa. A larger area under the curve (AUC) indicates a higher diagnostic accuracy. On the ROC curve, the point closest to the top left of the coordinate diagram is the critical value that reflects the highest sensitivity and specificity.
2. Confusion matrix: A confusion matrix is also called an error matrix, and it is a visual representation of the classification effect. A confusion matrix can be used to describe the relationship between the real category attribute of the sample data and the recognition result. It is a method for evaluating classifier performance and is widely used in pattern recognition. A confusion matrix is also a performance evaluation method that scholars often use when solving practical application problems.

### 4.5. Experimentation

**Figure 8:**
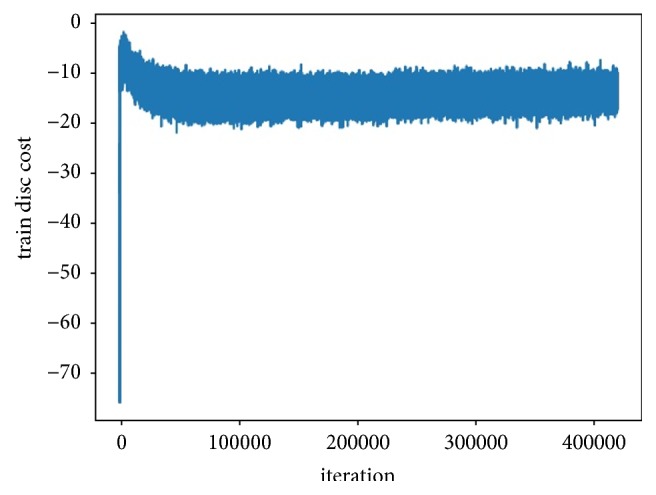
Training discrepancy cost of DCGAN.

From the LIDC-IDRI database, we acquired 590 noncentral calcification, 565 lobulation, 576 spiculation, 545 nonsolid/GGO texture sign patches, and 2,500 negative image patches. The experiment was conducted according to the following steps:

1. Set the initial values of T, $\hat{e}$, and *θ* to 6, 0.5, and 0.6, respectively.
2. Train the GAN until the discrepancy cost reaches a balance, as illustrated in Figure 8.
3. Train a primary fuzzy Co-forest based on the original samples, utilizing the features exported from the trained DCGAN discriminator in Step (2).
4. Input a random vector to DCGAN and transmit the generated features to the concomitant random fuzzy decision forest *E*_i_ in the primary Co-forest until $W_{i,t}\geq {\hat{e}}_{i,t-1}W_{i,t-1}/{\hat{e}}_{i,t}$ for all classes.
5. In this process, if the maximal weight of the fuzzy label from *E*_i_ exceeds the threshold *θ*, store both features with the matched label; otherwise, discard the generated image and generate a new image.
6. Retrain the corresponding tree *e*_i_ of *E*_i_.
7. Test the performance of the system.

As a comparison, we trained a C4.5 random forest according to the same scheme which has been used by G2C-CAD, as the baseline method.

We also conducted experiments on samples obtained from LISS.

## 5. Results

**Figure 9:**
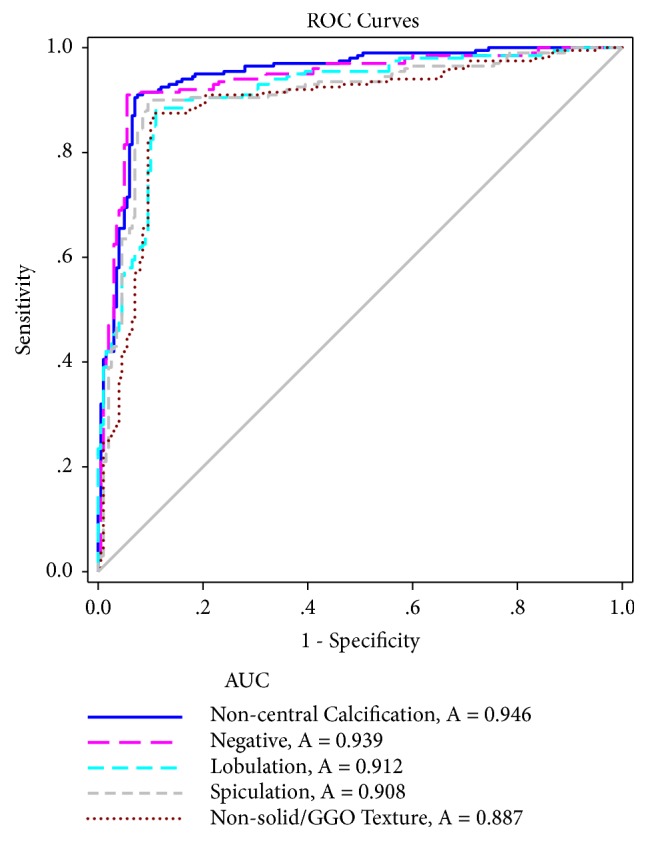
ROC curves for the 5 categories of sign ROI recognition results.

We divided the dataset into two parts, 90% for training and 10% for validation. In this way, the nodule distribution in the validation subsets is consistent with the nodule distribution of the original dataset according to the radiologists' consensus of their evaluations at the nodule level. The number of trees in the Co-forest classification model was 6, primary training was conducted on the training data, and finally, the system was further validated on the remaining 10%. The sensitivity and specificity of each class instance was calculated and compared using the ROC curves shown in Figure 9.

In Figure 9, the AUCs of noncentral calcification, negative image samples, lobulation, spiculation and nonsolid/GGO texture are 0.946, 0.939, 0.912, 0.908, and 0.887, respectively. From the curves in Figure 9, we can see that the G2C-CAD system achieves the highest overall classification accuracy on the calcification sign. Compared to noncentral calcification and negative image samples, the AUCs of lobulation, spiculation, and nonsolid/GGO texture are relatively lower.

**Figure 10:**
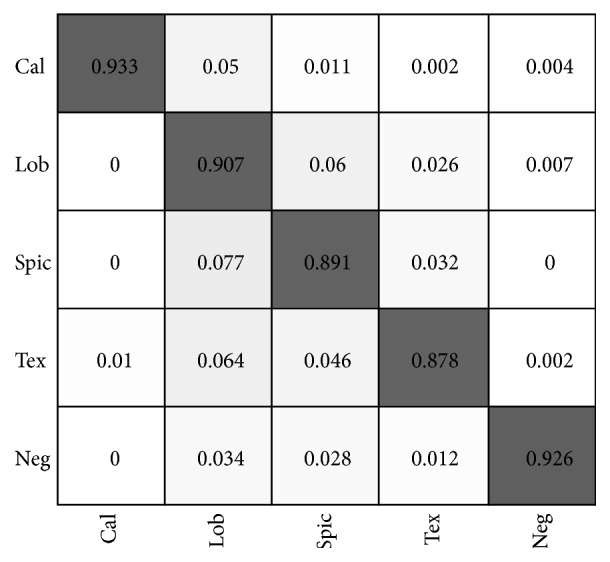
Confusion matrix of the predicted results.

To show the underlying classification error distribution, a confusion matrix of the 5 classification results is presented in Figure 10.

The diagonal numbers in the confusion matrix represent the recognition accuracy rates of the corresponding category, and the nondiagonal elements are misclassification rates, i.e., the ratio of other category test samples that were misclassified to this class. From Figure 10 we can see that the noncentral calcification sign has the highest classification accuracy, while the nonsolid/GGO texture sign has the highest misclassification rate. Misclassified spiculation signs are mostly recognized as lobulation. By comparing the test samples, we find that many of these two types of samples have very similar textures. From the confusion matrix, we can also see that most misclassifications occur between lobulation, spiculation, and nonsolid/GGO texture signs.

## 6. Discussion

**Figure 11:**
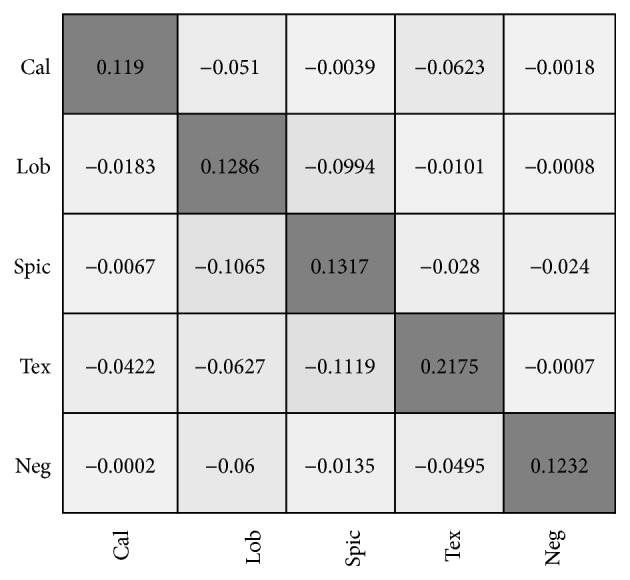
Performance difference matrix of G2C-CAD and C4.5.

We performed a performance comparison on each category between G2C-CAD and the C4.5 random forest model using the confusion matrix. First, we constructed confusion matrices reflecting the accuracies of G2C-CAD and C4.5. Then, we calculated the difference matrix for those two confusion matrices as shown in Figure 11. The float numbers on the diagonal represent the differences between G2C-CAD and C4.5 regarding their classification accuracy for the corresponding category, while the float numbers on the nondiagonal represent their differences in the classification error rate, showing the numbers for each class that were misclassified into the corresponding category.

Consequently, positive numbers on the diagonal and larger values indicate that G2C-CAD achieved a better performance than that of C4.5. For the elements that are not on the diagonal, the situation is the opposite. As shown in Figure 11, no positive values occur in the nondiagonal elements, which means that G2C-CAD possesses greater discrimination ability between each category. The element values on the diagonal are all positive, and the average value of the diagonal numbers in the difference confusion matrix is 0.144, demonstrating that our method has a better overall performance.

**Figure 12:**
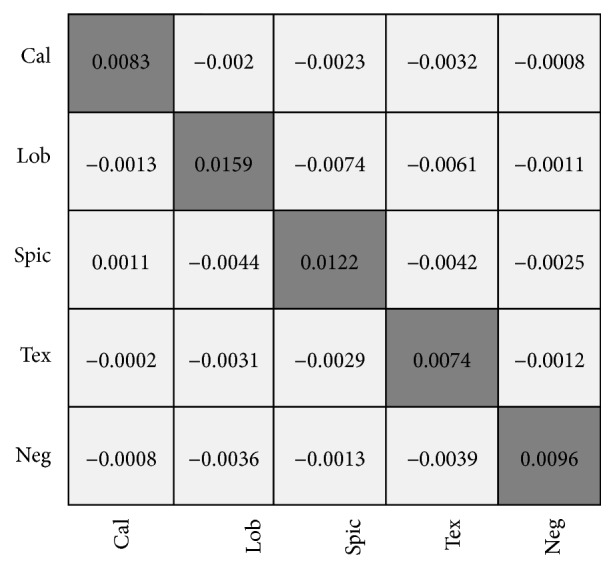
Performance difference matrix of fuzzy G2C-CAD and nonfuzzy G2C-CAD.

To verify the effectiveness of the fuzzy algorithm, we also conducted multiclassification experiments using G2C-CAD without the fuzzy algorithm and compared the performances of the two algorithms using a difference confusion matrix as shown in Figure 12.

From Figure 12, we can see that the discrimination performance of the CAD system employing the fuzzy algorithm is obviously better than that of the nonfuzzy one. Furthermore, as shown in Figure 12, the CAD performance of the fuzzy algorithm better distinguishes the difficult lobulation sign from the spiculation sign.

The experimental result on LISS shows higher performance; the areas under the ROC curve of GGO, lobulation, spiculation, pleural indentation, and negative image samples are 0.972, 0.964, 0.941, 0.967, and 0.953, respectively. By comparing the training dataset and test samples, we found that the main reason may be that the samples in different categories are more separable visually.

## 7. Conclusion

In this paper, by coupling a GAN with a semisupervised learning approach, we proposed a G2C-CAD method to detect signs that are highly correlated with malignant pulmonary nodules. We first trained a DCGAN on a small sample set. Then, we extracted the features from the CNN discriminator trained with the training samples and used them to train a primary fuzzy Co-forest classifier. Then, we use the trained DCGAN to generate large amounts of realistic fake samples. Based on these fake samples, we conducted semisupervised learning with the fuzzy Co-forest and finally obtained a classifier with excellent performance. By validating on the LIDC dataset, the area under the ROC curve for five sign types, noncentral calcification, negative image samples, lobulation, spiculation, and nonsolid/GGO texture, reached 0.946, 0.939, 0.912, 0.908, and 0.887, respectively. On the LISS dataset, the proposed system showed comprehensively higher classification performances than those of a trained C4.5 classifier. The experimental results show that the proposed G2C-CAD is an appropriate method for solving the problem of insufficient samples in the medical image analysis field. Moreover, our system can also be used to establish a training sample library for CAD classification diagnosis, which holds great significance for future medical image analysis.

In future work, we plan to combine this method with multiple-instance learning to perform weak supervised learning directly on CT slice images or to extend the algorithm, making it suitable for use in the 3D medical image classification field.

## Figures and Tables

**Algorithm 1:**
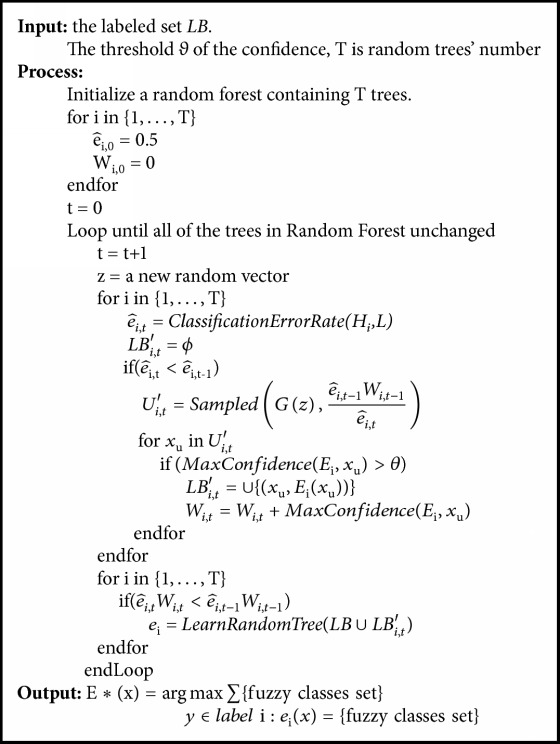
G2C-CAD.

**Table 1:** The scheme of sign selection.

	Used as signs	Abandoned
Subtlety	-	-
Internal structure	-	-
Calcification	=4	<>4
Sphericity	-	-
Margin	-	-
Lobulation	>=4	<4
Spiculation	>=4	<4
Texture	<=2	>2

**Table 2:** Sign samples of calcification, lobulation, spiculation, and nonsolid/GGO texture.

	Noncentral calcification		Lobulation		Spiculation		Nonsolid/GGO texture	
	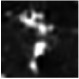	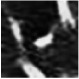	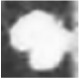	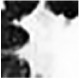	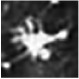	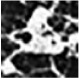	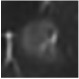	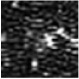
Subtlety	4.67	5.0	5.0	4.0	4.0	5.0	3.67	3.0
Internal structure	1.0	1.0	1.0	1.0	1.0	1.0	1.0	1.0
Calcification	**4.0**	**4.0**	6.0	6.0	6.0	6.0	6.0	6.0
Sphericity	3.33	2.67	4.25	4.0	5.0	5.0	4.33	4.0
Margin	3.67	4.67	4.5	4.0	2.0	4.0	2.67	1.0
Lobulation	1.67	2.0	**4.25**	**4.0**	4.0	1.0	2.67	1.0
Spiculation	1.33	1.33	2.25	4.0	**4.0**	**5.0**	1.67	2.0
Texture	5.0	4.67	4.75	4.0	5.0	4.0	**1.33**	**1.0**

**Table 3:** Number of selected instances of LISS.

CISL Category	# of Lesion Regions
GGO	45
Lobulation	41
Spiculation	29
Pleural Indentation	45
Negative	80

## Data Availability

The data used to support the findings of this study are available from the corresponding author upon request.
